# The Significant Antioxidant Effect Exerted by Pomegranate (*Punica granatum*): The Hidden Polyphenols

**DOI:** 10.3390/antiox15030276

**Published:** 2026-02-24

**Authors:** Rosamaria Caminiti, Valeria Mazza, Jessica Maiuolo, Federico Liuzzi, Francesca Oppedisano, Saverio Nucera, Salvatore Ragusa, Luigi Tucci, Giuseppe Trunfio, Lucia Carmela Passacatini, Sara Ilari, Ernesto Palma, Vincenzo Mollace, Carolina Muscoli

**Affiliations:** 1 Department of Health Science Institute of Research for Food Safety & Health (IRC-FSH), University “Magna Graecia” of Catanzaro, 88100 Catanzaro, Italy; r.caminiti@unicz.it (R.C.); valeria.mazza@unicz.it (V.M.); oppedisanof@libero.it (F.O.); saverio.nucera@hotmail.it (S.N.); l.tucci@head-sa.com (L.T.); g.trunfio@head-sa.com (G.T.); palma@unicz.it (E.P.); mollace@libero.it (V.M.); muscoli@unicz.it (C.M.); 2 Laboratory for Techniques and Processes in Biorefineries, ENEA—Trisaia Research Centre, S.S. Jonica 106, Km 419+500, 75026 Rotondella, Italy; federico.liuzzi@enea.it; 3 PLANTA/Research, Documentation and Training Center, 90121 Palermo, Italy; ragusa@unicz.it; 4 Laboratory of Physiology and Pharmacology of Pain, IRCCS San Raffaele Roma, 00166 Rome, Italy; carmela.passacatini@sanraffaele.it (L.C.P.); sara.ilari@sanraffaele.it (S.I.); 5 Department of Health Sciences, University of Catanzaro Magna Græcia, CISVetSUA, 88100 Catanzaro, Italy

**Keywords:** dietary fibre, polyphenols, antioxidant properties, neurodegeneration, neurons, astrocytes, non-extractable polyphenols

## Abstract

**Background**: Although the definition of dietary fibre is complex and constantly evolving, today we can identify it as “carbohydrate polymers with at least 10 monomeric units, which are not hydrolysed in the small intestine of humans”. In addition to the numerous and well-known benefits of dietary fibre for human health, our attention is drawn to its antioxidant properties, achieved through polyphenolic compounds linked to polysaccharide complexes. This study investigated the antioxidant effects of an extract from the fruit of *Punica granatum* (PUN), particularly rich in polyphenols, fibre, flavonoids, vitamins, organic acids, minerals, amino acids, and alkaloids. Furthermore, these effects were evaluated in two human nervous system cell lines under oxidative stress induced by hydrogen peroxide. **Methodology**: After examining the fibre composition, some polyphenols present in the extract were identified and quantified by HPLC. Furthermore, the antioxidant power of PUN was measured using the DPPH method, the chelating activity assay, the reducing power test, the ORAC method, the measurement of reactive oxygen species accumulation, the quantification of lipid peroxidation, and the detection of mitochondrial superoxide in cell cultures. **Results**: The results were consistent, and PUN demonstrated a strong antioxidant potential, justified not only by the high content of easily extractable polyphenols (EPPs) but also by a further addition of these more difficult to identify compounds (NEPPs), indicated as “hidden polyphenols”; therefore, the total polyphenol content in the extract resulted from the sum of EPPs + NEPPs (71 ± 7.9 + 55 ± 6.4 mg = 126 ± 14.3 mg gallic acid equivalent (GAE)/g dry weight). The fraction of hidden polyphenols could therefore explain a mechanism by which the fibre exerts an antioxidant effect. Another important result was achieved by the cell lines used, both of which were significantly protected by PUN following oxidative damage generated by a pro-oxidant treatment. However, astrocytes were found to be more responsive and sensitive than were human neurons. At the same time, PUN mitigated the effects of oxidative damage, and it could be hypothesised that this extract could be used to extinguish the A1 phenotype. **Conclusions**: We can conclude that the fibrous component of pomegranate is related to the antioxidant property exerted, and the neurodegeneration caused by oxidative stress could be slowed following the intake of *Punica granatum*. It is possible to identify the pomegranate as a “superfood” or “functional food”, with excellent nutritional characteristics and chemical composition.

## 1. Introduction

Dietary fibre is a component of plant-derived foods; it is resistant to digestion in the small intestine and only partially digestible in the colon, thanks to the enzymes of the gut microbiota [1]. The first distinction of dietary fibre is based on its chemical composition: the two major classes are polysaccharides of non-starchy origin and lignin. Polysaccharides are polymers produced by the aggregation of monosaccharide molecules. Cellulose, the most abundant molecule in nature, is a linear polymer of 1,4β-linked glucose units, and the chain confers a myofibril crystalline structure [2]. In addition to cellulose, the group of polysaccharides of non-starchy origin includes a group of substances collectively called non-cellulosic polysaccharides which comprise a large number of heteroglycans, such as hemicellulose, pectic substances, mucilages, gums, and algal polysaccharides. Hemicellulose contains β-1,4-linked pyranoside sugars; it is usually branched and smaller than cellulose [3]. Pectic substances are polysaccharides in which D-galacturonic acid is a main constituent. They are structural components that have the function of intercalating between cells, cementing and fortifying the cell wall structure [4]. The second class consists of lignin, which is not a polysaccharide but a complex and heavy organic polymer composed primarily of phenolic compounds. It is formed of a polymeric structure of phenylpropane units, a class of aromatic compounds. Its main function is to provide rigidity to cell walls and allow connections between cells, creating a material that is very resistant to bending, impacts, and compression. Furthermore, lignified tissues prevent the attack of microorganisms and the penetration of enzymes destructive to the cell wall [5]. Lignin also makes cells impermeable, thanks to its hydrophobicity. Cells that provide support to the plant and ensure the transport of water and mineral salts have cell walls impregnated with lignin [6]. The second characteristic that differentiates fibres is their solubility in water, which influences their metabolism and digestibility. Among the soluble fibres are pectins, gums, and mucilages, while the insolubles comprise cellulose, hemicellulose, and lignin [7].

Fibre exhibits various beneficial properties, including the following:

- A prebiotic effect: it stimulates the enrichment of the composition of the intestinal microbiota [8];
- The lowering of glucose and cholesterol levels in the blood: fibre results in a satiating effect that slows down the passage of food inside the digestive tract and the absorption of carbohydrates and fats [9];
- The improvement of intestinal function, promoting the elimination of unabsorbed food, avoiding stagnation of faecal mass, and reducing disorders including irritable bowel syndrome, inflammatory bowel diseases, constipation, and diverticular disease [10];
- The maintenance of gastrointestinal health, including the reduction in the development of colorectal and lung malignancies and a slowing of the progression of neurodegenerative diseases [11];
- The antioxidant role of fibre has also emerged in recent years [12]. This effect could be justified by the hydrogen bonding of carbohydrates with polyphenols, which would facilitate the neutralisation of free radicals through the donation of additional electrons [13]. These polyphenols could prevent cellular damage and act as a protective barrier against chronic diseases associated with oxidative stress [14].

Thanks to its ability to sustain and enhance health and lower disease risk, fibre is regarded as an excellent supplement to add to foods to boost their nutrition [15].

The pomegranate (*Punica granatum*) is a plant belonging to the *Lythraceae* family, native to Southwest Asia. It is currently cultivated in this area (Caucasus, Azerbaijan, Armenia, Iran, Afghanistan, Uzbekistan, Turkey, Palestine, Arabia, India, Indonesia, Malaysia, and Pakistan), and it has been present in the entire Mediterranean area since ancient times. It is also cultivated in the arid regions of tropical Africa [16]. *Punica granatum* prefers a mild climate, characterised by cool winters and hot summers, but is extremely drought-tolerant [17]. The plant appears as a tree with a bushy and deciduous appearance, capable of reaching 6–7 m in height and living for over 100 years. The leaves are opposite, narrow, elongated, and shiny, measuring 2 cm in width and 4–7 cm in length. The flowers are a bright red colour, approximately 3 cm in diameter, and have three to four petals, depending on the variety. The fruit is a round to slightly elongated berry with a hard, leathery peel, measuring 5 to 12 cm in diameter. The fruit has several robust internal partitions and contains seeds, known as arils [18]. The seeds are surrounded by a translucent pulp that ranges from white to ruby red and is acidic, edible, sweet, and fragrant [19]. The pomegranate is considered an exceptional fruit, as it contains many compounds beneficial to health, such as polyphenols, fibre, flavonoids, vitamins, organic acids, minerals, amino acids, and alkaloids [20]. These compounds are responsible not only for antioxidant, anti-inflammatory, anticancer, antiviral, and antibacterial properties but also for protective metabolic effects (against diabetes, hypertension, and hyperlipidemia) [21]. The chemical components mentioned are present in every part of the fruit, but particularly in the peel and other non-edible parts [22,23].

Since pomegranate is made up of a fibrous component, it would be interesting to test the effect of the fruit extract on oxidative stress. Therefore, this article measured the antioxidant potential of the extract and tested it on an in vitro model comprising two human cell lines: SH-SY5Y neurons and U373-MG astrocytes. Neurons are the fundamental cells of the nervous system, and since they cannot replace themselves, any damage causes them to degenerate, resulting in the loss of motor or cognitive functions [24,25]. Astrocytes are glial cells that support neurons in the structure, metabolism, and regulation of synapses [26]. They are essential for learning, memory and proper brain function; their malfunction is implicated in several neurological pathologies [27]. Neurons and astrocytes were exposed to hydrogen peroxide (H_2_O_2_), responsible for the accumulation of reactive oxygen species (ROS). Oxidative stress is one of the innumerable causes of neurodegeneration onset, and is a recurring phenomenon in many neurodegenerative diseases [28,29]. This study aims to understand whether the antioxidant effect exerted by pomegranate is related to its fibrous portion and to inform the public that neurodegeneration caused by oxidative stress may be slowed following the intake of *Punica granatum*.

## 2. Materials and Methods

### 2.1. Plant Material and Sample Preparation

The harvesting of fruit and its taxonomic identification have been described by the authors of [30]. Mature fruits were washed twice with filtered water to remove particulate matter and surface residues. Whole fruits were mechanically pressed to obtain crude juice, which was separated from the solid fraction by centrifugation. The clarified juice was further filtered and subjected to purification on a column packed with Sepabeads SP700 polymeric adsorbent resin (Mitsubishi Chemical Corporation, Tokyo, Japan). After sample loading, the aqueous phase was discarded, and phenolic compounds retained on the resin were eluted with 80% (*v*/*v*) ethanol (Sigma Aldrich, Milan, Italy). The ethanol eluate, enriched in bioactive constituents, was concentrated under vacuum at a controlled temperature (<45 °C) to obtain a liquid extract. The concentrated extract was subsequently dried by spray drying to yield a fine powder, and 10 mg were dissolved in 1 mL of DMSO [27]. The extract has been indicated as PUN.

### 2.2. Fibre Analysis

Analysis of ligno-cellulosic materials for sugars, lignin, and ash content was evaluated using the NREL protocol described by Sluiter et al. [31]. Carbohydrate content was determined using a two-step acid hydrolysis method. Initially, to decompose polysaccharides into oligomers, the sample was treated with 72% sulfuric acid at 30 °C for 1 h. Subsequently, the product was treated with 3% sulfuric acid at 121 °C for 1 h in an autoclave to ensure complete conversion of oligomers into monomers. These monomers were then analysed using ionic chromatography (HPIC) with a DIONEX DX300 chromatograph (UltiMate 3000, Thermo Fisher Scientific Corporation, Milan, Italy), a Nucleogel Ion 300 OA column (MACHEREY-NAGEL, Düren, Germany), an ED50 refractive index detector (Thermo Fisher Scientific Corporation, Milan, Italy), and 0.05 M H_2_SO_4_ as the mobile phase (40 °C, 0.4 mL/min). The acid-insoluble lignin content was measured gravimetrically by filtration of the residue with Whatman GFA filters. Acid-soluble lignin was quantified using a Varian Cary 500 spectrophotometer (SpectraLab Scientific Inc., Markham, ON, Canada) at a 205 nm wavelength. The ash content was evaluated by placing the sample in an oven at 575 °C overnight. The characterisation process was carried out in triplicate and represents the mean value with the standard deviation.

### 2.3. DPPH Assay

The antioxidant activity of PUN was measured using the stable radical 2,2-diphenyl-1-picrylhydrazyl (DPPH, Sigma Aldrich, Milan, Italy), and the reduction in absorbance was measured. Experimentally, 850 µL of the DPPH solution was added to 50 µL of various extract concentrations (0–100 µg/mL), keeping the mixture in the dark for 20 min. Subsequently, the absorbance was read by a UV–Vis spectrophotometer (Multiskan GO, Thermo Scientific, Denver, CO, USA) at 517 nm at room temperature. The reduction in absorbance was visible as a change in colour from purple to yellow. The results obtained were expressed as % inhibition value and IC_50_. The latter represents the concentration of extract needed to remove 50% of radicals.

### 2.4. Oxygen Radical Absorbance Capacity (ORAC) Assay

The antioxidant capacity of PUN was evaluated by measuring the transfer of hydrogen atoms and by monitoring the loss of fluorescence of the fluorescein probe over time. The fluorescence reaction is generated spontaneously by the degradation of 2,2-azobis (2-methylpropionamidine) dihydrochloride (AAPH, Sigma Aldrich, Milan, Italy) at 37 °C. The peroxyl radical oxidises the fluorescein, causing a gradual loss of the fluorescent signal. 6-Hydroxy-2,5,7,8-tetramethylchroman-2-carboxylic acid (Trolox, Sigma Aldrich, Milan, Italy) inhibits fluorescence decay. A Trolox dose–response curve (15.25, 30.5, and 61 μg/mL) was constructed, while the extract was used at 100 μg/mL. Fluorescein fluorescence decay was measured using a microplate reader at wavelengths of 485 and 520 nm for excitation and emission, respectively. The measurements were taken in triplicate, every 2 min for 1.5 h, and the data obtained from the fluorescence curves over time showed the average antioxidant efficacy of the samples. A regression equation was constructed by comparing the net area below the fluorescein decay curve and the Trolox concentration. The data obtained showed the antioxidant efficacy of the samples.

### 2.5. Reducing Power Assay

The reducing power of PUN was measured by the ability to transform Fe^3+^ into Fe^2+^ through a spectrophotometric assay, as described in [32]. Ascorbic acid (Sigma Aldrich, Milan, Italy) was used as a standard at different concentrations (0.01–0.32 mg/mL). Both standard and PUN were added to 1.0 mL of deionised water, 2.5 mL of phosphate buffer at pH 6.6, and 2.5 mL of potassium ferricyanide (1%). These solutions were incubated at 50 °C for 20 min. Then, 2.5 mL of trichloroacetic acid (Sigma Aldrich, Milan, Italy) (10%) was added, and the solutions were thoroughly mixed and centrifuged at 3000 rpm for 10 min. The supernatant of each sample was mixed with 2.5 mL of distilled water and 0.5 mL of a freshly prepared 0.1% ferric chloride solution. The absorbance of the mixture was read at 700 nm. An increase in absorbance of the reaction mixture indicated an increase in reducing power.

### 2.6. Ferrous Ion (Fe^2+^) Chelating Activity Assay

The antioxidant potential of a compound can be assessed by measuring the formation of the Fe^2+^–ferrozine complex. Ascorbic acid (Sigma Aldrich, Milan, Italy) was used as a reference standard, and the reaction was tested in the concentration range of 0.0625–2 mg/mL. A total of 1 mL of PUN was mixed with 1 mL of methanol, 0.1 mL of 2 mM FeCl_2_, and 0.2 mL of 5 mM ferrozine (Sigma Aldrich, Milan, Italy). The resulting solutions were kept in the dark at room temperature for 10 min, and absorbance was measured at 562 nm. The chelating activity is the antioxidant potential of a compound, evaluated by measuring the formation of the Fe^2+^–ferrozine complex, and the results are reported as a percentage of inhibition of its formation.

### 2.7. Cell Cultures

In vitro experiments were conducted on two human cell lines: neuroblastoma cells (SH-SY5Y) and astrocytoma cells (U373-MG). Both lines were purchased from the American Type Culture Collection (ATCC; U373-MG: CVCL-2219; SH-SY5Y: CRL-2266) and maintained in culture in Eagle’s Minimum Essential Medium (Sigma Aldrich, Milan, Italy) supplemented with 10% fetal bovine serum (Thermo Fisher Scientific, Milan, Italy), non-essential amino acids, penicillin (100 IU/mL), and streptomycin (100 μg/mL) (Sigma Aldrich, Milan, Italy). When the cells reached 50% confluence, they were treated with PUN at 100 µg/mL for 24 h and then exposed to hydrogen peroxide (H_2_O_2_, Sigma Aldrich, Milan, Italy, 200 μM,) for 20 min.

### 2.8. Cell Death

Both cell lines were seeded in 35 mm 6-well plates at a density of 20 × 10^4^. Twenty-four hours after plating, the growth medium was replaced with fresh normal medium (control cultures) or with medium supplemented with PUN (10–200 µg/mL) for 24 h. Cell viability was assessed by cell exclusion of trypan blue (0.4% *w*/*v*, Sigma Aldrich, Milan, Italy); this dye penetrates damaged or dead cells because they have a damaged membrane, so that they appear stained, while it is excluded from viable cells with the membrane intact, and these appear unstained. Cell death was reported as the percentage of stained (non-viable) vs. total cells counted.

### 2.9. ROS Accumulation Measurement

The ROS measurement test was performed under both fluorimetry and cyto-fluorimetry, obtaining the same results. Experimentally, the fluorescein molecule (H_2_DCF-DA, Invitrogen, Thermo Fisher Scientific, Milan, Italy) has been used, which easily penetrates cells and is cleaved by intracellular esterases to form H_2_DCF. This compound can no longer leave the cells and binds to the ROS present. H_2_DCF is transformed into DCF, which is highly fluorescent. DCF quantification is proportional to ROS content. Specifically, cells were seeded in 96-well microplates with a density of 2 × 10^4^ cells/well or in 6-well microplates with a density of 8 × 10^4^ cells/well. The next day, the cells were treated with PUN, as described. After 24 h, the medium was replaced with fresh medium containing H_2_DCF-DA (25 μM) for 30 min. At the end of the exposure time, the cells were washed with PBS and treated or not with H_2_O_2_ (200 µM, 20 min). Finally, the cells were subjected to fluorimetric (Fluoroskan Ascent, Thermo Scientific) or flow cytometric analysis (FACS Accury, Becton Dickinson, Franklin Lakes, NJ, USA).

### 2.10. Malondialdehyde Assay

Malondialdehyde (MDA) detection was performed by measuring the thiobarbituric acid reactive substances (TBARS, Sigma Aldrich, Milan, Italy). Lysate, after interaction with 10% NaOH, 20% acetic acid, and TBA, was boiled at 95° C for 1 h and placed on ice for 10 min. Then, the samples were centrifuged at 1600× *g* (10 min at 4° C), loaded into a black 96-well microtiter plate, and fluorometrically measured at an excitation of 530 nm and emission of 550 nm.

### 2.11. Detection of Mitochondrial Superoxide

To evaluate the presence of mitochondrial superoxide, a fluorogenic dye (MitoSOX™ Red, Invitrogen by Thermo Fisher Scientific, Milan, Italy) was used. These indicators are highly selective and, after entering viable cells, reach the mitochondria. Within these organelles, the reagent is oxidised by the superoxide, emitting a red-bright fluorescence that can be observed and measured. Oxidation of the fluorescent probe can only be carried out by superoxide, but not by other reactive species (oxygen or nitrogen). Experimentally, 5 × 10^4^ cells were seeded per well in a 96-well plate. After carrying out the treatments as described (hydrogen peroxide or PUN + hydrogen peroxide), 100 μL of fluorescent probe per well was added to cover the adhering cells. Cells were incubated for 30 min at 37 °C and 5% CO_2_. After the prescribed time, the cells were washed three times with a heated buffer (HBSS with calcium and magnesium), and the fluorescence was evaluated within two hours of staining. Fluorescence was detected through a fluorimeter (Fluoroskan Ascent, Thermo Scientific) and observed on cell lines using an Evos M5000 device (Invitrogen by Thermo Fisher Scientific).

### 2.12. Determination of Total Phenolic and Flavonoid Content

PUN extract is rich in polyphenols and flavonoids, and its content was calculated using the Folin–Ciocalteu colourimetric assay and the aluminium chloride assay (Sigma Aldrich, Milan, Italy), respectively. For the quantification of polyphenols, we used several solutions of gallic acid (Sigma Aldrich, Milan, Italy). A total of 400 µL of PUN was mixed with 0.8 mL of 10 times diluted Folin–Ciocalteu reagent. A total of 0.8 mL of 7% (*w*/*v*) sodium carbonate was added to the samples, and the resulting solution was allowed to stand for two additional hours under constant stirring. Absorbance was measured at 760 nm, and the total phenolic content of the extracts was expressed in mg gallic acid equivalent (GAE)/g dry weight. For quantitation of flavonoids, 1 mL of extract was mixed with 1 mL of 2% aluminium chloride in methanol. After 30 min, the absorbance was read at 430 nm, and the results were expressed in mg quercetin equivalent (mg QE/g extract)/g dry weight.

### 2.13. HPLC–UV Analysis

Chromatographic analyses were performed on a PerkinElmer HPLC system with UV/PDA detection (Shimadzu Corporation, Milan, Italy). A reversed-phase C18 column (250 × 4.6 mm, 5 µm particle size) was used for all separations. Two complementary acquisition methods were employed: one targeted to hydrolysable tannins (punicalagins and ellagic acid) monitored at 360 nm and one dedicated to anthocyanins monitored at 520 nm. The system was controlled, and data was processed using Chromera software (version 4.0). Samples were filtered through 0.45 µm PTFE syringe filters before injection. Chromatographic conditions comprised a column temperature of 30 °C, a flow rate of 1.0 mL·min^−1^, and an injection volume of 10 µL. Mobile phase A consisted of water with 0.1% (*v*/*v*) trifluoroacetic acid for tannins, water with 0.3% (*v*/*v*) for anthocyanins, and mobile phase B consisted of acetonitrile.

Hydrolysable tannins: punicalagins method (λ = 360 nm). The gradient was optimised to resolve punicalagin isomers and ellagic acid as follows: 0–10 min, 15% B; 10–30 min, linear to 25% B; 30–32 min, linear to 100% B; 32–34 min, return to 15% B and re-equilibration until 40 min total runtime. Under these conditions, the major tannin peaks were observed at retention times consistent with those in the sample report: punicalagin A ~10 min, punicalagin B ~12 min, and ellagic acid ~21 min.

Anthocyanins method: (λ = 520 nm). To separate the more polar anthocyanin glycosides, the following gradient was used: 0–4 min, 5% B; 4–14 min, linear to 10% B; 14–30 min, linear to 15% B; 30–32 min, linear to 90% B; 32–37 min, return to 5% B and re-equilibration to 40 min total runtime. Major anthocyanin peaks identified in the sample chromatogram appeared at retention times corresponding to those for delphinidin-3,5-diglucoside (~20.94 min), delphinidin-*3*-*O*-glucoside (~26.6 min), cyanidin-3,5-diglucoside (~24 min), and cyanidin-3-*O*-glucoside (~30.5 min). Total anthocyanin area was reported and used for quantification. Quantification was performed by external standard calibration. Standards of punicalagin A, punicalagin B, and cyanidin-3-*O*-glucoside were purchased from Sigma-Aldrich and were used to construct six-point calibration curves covering the expected concentration range.

### 2.14. Determination of Non-Extractable Polyphenols (NEPPs)

To determine the NEPP, 0.25 g of extract was obtained and blended with a solution of methanol (Sigma Aldrich, Milan, Italy) and water acidified with 1% HCl (50/50, *v*/*v*, Sigma Aldrich, Milan, Italy) to achieve a pH close to 2. The samples were homogenised in an orbital shaker and subsequently centrifuged (4500× *g*, 10 min, room temperature). This process was repeated twice using a solution of acetone/water (70/30 *v*/*v*, Sigma Aldrich, Milan, Italy). The pellet was mixed with 10 mL of methanol/water/formic acid (79/19/1, *v*/*v*/*v*), which underwent a 20 h incubation at 85 °C, followed by centrifugation (4500× *g*, 10 min, room temperature). The collected supernatant was isolated [33], and the total phenolic content was analysed using the described Folin–Ciocalteu method.

## 3. Results

### 3.1. Fibrous Profile of PUN

**Figure 1:**
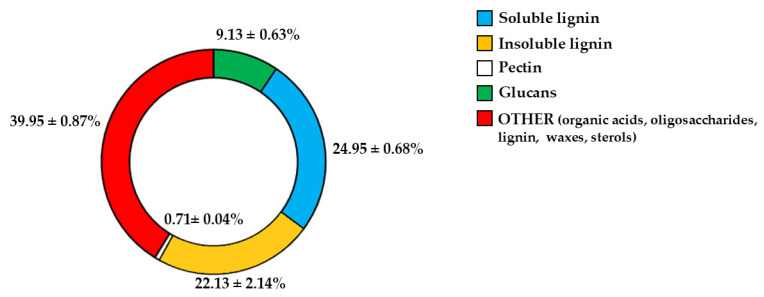
Fibrous profile of PUN. Figure 1 shows the percentages of the fibrous fractions present in PUN.

Since fibre is among the constituents of pomegranate [11,31], we wanted to determine which fibrous components were present in PUN. The respective results are represented in Figure 1. As shown, the beta-glucans are represented at 9.13% and are markedly outperformed by lignin (soluble = 24.95%; insoluble = 22.13%). Pectin is present in modest quantities, equal to 0.71%. It is interesting to note that a preponderant percentage (39.95) is identified as “OTHER”, which can be represented by phenolic acids, proteins, oligosaccharides of various origins, lignin, waxes, and sterols.

### 3.2. Antioxidant Effects of PUN

**Figure 2:**
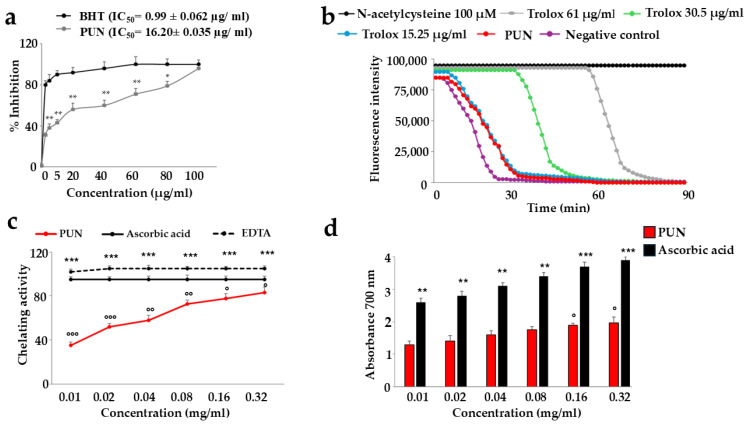
Antioxidant ability of PUN. Panel (**a**): Butylated hydroxytoluene (BHT) is a synthetic antioxidant used as a standard because it is stable and easily available. The relevant IC_50_s can easily be viewed from the curves (BHT and PUN). * denotes *p* < 0.05 vs. the respective concentration of the BHT curve. ** denotes *p* < 0.01 vs. the respective concentration of the BHT curve. A Tukey–Kramer comparison test followed the analysis of variance (ANOVA). Panel (**b**): The red curve represents the oxygen radical absorption capacity of PUN. Three independent experiments were performed, but the graph of a representative experiment is shown. Panel (**c**): The chelating activity assay was measured. EDTA was used as a positive control. Three independent experiments were performed, and the values are expressed as the mean ± SD. *** denotes *p* < 0.001 vs. the concentration of PUN 0.01 mg/mL; ° denotes *p* < 0.05 vs. EDTA at the same concentration; °° denotes *p* < 0.01 vs. EDTA at the same concentration; °°° denotes *p* < 0.001 vs. EDTA at the same concentration. A Tukey–Kramer comparison test followed the analysis of variance (ANOVA). In panel (**d**), the reducing power assay is shown, and ascorbic acid was used as a reference standard. Six independent experiments were carried out, and the values are expressed as the mean ± SD. ** denotes *p* < 0.01 vs. the concentration of PUN 0.01 mg/mL. *** denotes *p* < 0.001 vs. the concentration of PUN 0.01 mg/mL. ° denotes *p* < 0.05 vs. the concentration of PUN 0.01 mg/mL. A Tukey–Kramer comparison test followed the analysis of variance (ANOVA).

The PUN extract demonstrated a relevant antioxidant effect, as indicated in Figure 2. Panel a shows the concentration required to inhibit the accumulation of reactive oxidant species by 50%. The IC_50_ of PUN was equal to 16.20 ± 0.035 µg/mL. Panel b reports the way in which PUN manages to inhibit the activity of free radicals. It is interesting to observe that this effect is comparable to that measured by Trolox at 15.25 µg/mL, and the specific ORAC value of PUN is 5.8 µmol Trolox equivalents (TE) per g of extract. In panel c, the chelating activity of PUN is highlighted, while in panel d, the reducing potential is shown. In both latter assays, as the PUN concentration increases, the chelating power and the reducing power also significantly increase.

### 3.3. Effects of PUN on Cell Viability

**Figure 3:**
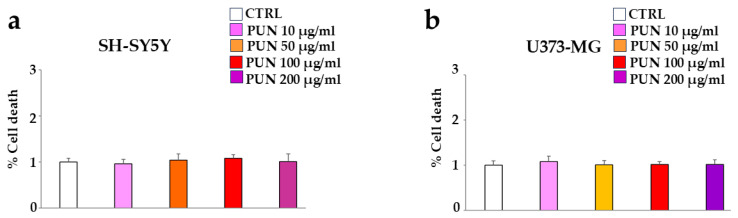
PUN effects on the vitality of cell lines. CTRL corresponds to cells not treated with PUN but exposed to vehicle (DMSO) in which the extract is dissolved. The value of the control (vehicle) was arbitrarily set equal to 1, and the other values were related to it. Panel (**a**,**b**) show the cell death of neurons and astrocytes following treatment with different concentrations of PUN, respectively.

First of all, we studied the effect of PUN on the considered cell lines, human neurons (SH-SY5Y), and astrocytes (U373-MG). To choose the suitable dose of PUN to use, we tested different concentrations on both cell lines (10–200 µg/mL), and since none showed significant variations compared to the control, we arbitrarily chose a concentration of 100 µg/mL, which was neither too low and ineffective nor too high and toxic. These results are represented in panels a and b of Figure 3, respectively.

### 3.4. Antioxidant Effects of PUN on Cell Lines

**Figure 4:**
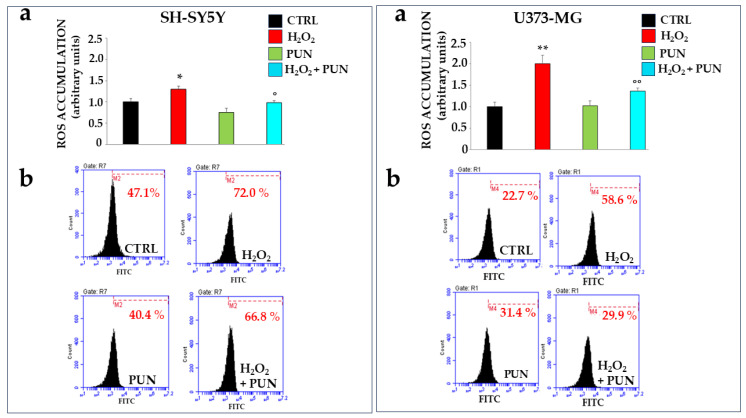
Antioxidant effects of PUN on cell lines. Two boxes are shown in the figure: the one on the (**left**) represents neurons, and the one on the (**right**) represents astrocytes, as indicated. Panel (**a**) shows the ROS produced, measured fluorimetrically. The value of the control (untreated cells) was arbitrarily set to 1, and the other values were derived from it. Astrocytes appear more susceptible to oxidative damage than are neurons; in fact, treatment with hydrogen peroxide resulted in the accumulation of 65% more ROS compared to that of neurons. Three independent experiments were performed, and the values are expressed as the mean ± SD. * denotes *p* < 0.05 vs. the CTRL. ** denotes *p* < 0.01 vs. CTRL. ° denotes *p* < 0.05 vs. H_2_O_2_. °° denotes *p* < 0.01 vs. H_2_O_2_. A Tukey–Kramer comparison test followed the analysis of variance (ANOVA). The ROS produced, measured flow cytometrically, are expressed in panel (**b**) of both boxes. Three independent experiments were performed, but the graph of only one representative experiment is highlighted. As can be observed, each plot is accompanied by a marker (M2 in neurons and M4 in astrocytes), which is arbitrarily drawn in the CTRL and remains unchanged in all other plots. The marker identifies the cells in that region of space and is represented by a percentage displayed below the marker, calculated by the flow cytometer. The comparison of percentages comprises the results of the experiment. The results in panel (**a**) have the same trend as the results reported in panel (**b**).

The antioxidant effect of PUN has also been investigated using two cell lines exposed to hydrogen peroxide. Specifically, neurons and astrocytes were treated with PUN at 100 µg/mL for 24 h and subsequently exposed to hydrogen peroxide (H_2_O_2_, 200 μM) for 20 min. Hydrogen peroxide is responsible for the production and accumulation of ROS. As shown in Figure 4, PUN reduced radical species. The box on the left refers to neurons SH-SY5Y, while the one on the right is dedicated to astrocytes U373-MG. In panel a, the ROS levels in both boxes were measured using fluorimetry. When both cell lines were exposed to hydrogen peroxide, ROS increased significantly compared to the results for untreated cells. Treatment with PUN alone did not lead to any accumulation of reactive species, highlighting that the extract lacks an intrinsic oxidant potential. Finally, a pre-treatment with PUN, followed by exposure to hydrogen peroxide, highlighted a significant reduction in the accumulation of ROS compared to the results for the treatment with hydrogen peroxide alone. In particular, astrocytes are more susceptible to oxidative damage than are neurons, accumulating a higher percentage of ROS, about 65%, than the results for neurons. In panel b, of both boxes, results similar to those already described are obtained through flow cytometric analysis. Finally, in panel c of both boxes, the quantification of data shown in panel b is highlighted.

**Figure 5:**
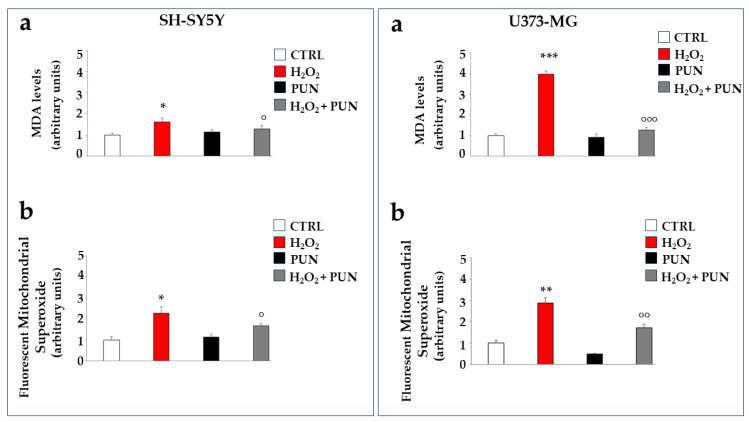
Antioxidant effects of PUN on cell lines. Two boxes are shown in the figure: the one on the (**left**) represents neurons, and the one on the (**right**) represents astrocytes, as indicated. Panel (**a**) shows the MDA level produced, measured fluorimetrically. The value of the control (vehicle) was arbitrarily set to 1, and the other values were derived from it. Hydrogen peroxide induces 41% higher MDA accumulation in astrocytes than in neurons. Three independent experiments were performed, and the values are expressed as the mean ± SD. * denotes *p* < 0.05 vs. the CTRL. *** denotes *p* < 0.001 vs. the CTRL. ° denotes *p* < 0.05 vs. H_2_O_2_. °°° denotes *p* < 0.001 vs. H_2_O_2_. A Tukey–Kramer comparison test followed the analysis of variance (ANOVA). Panel (**b**) of both boxes highlights the levels of mitochondrial superoxide, formed and accumulated, following the treatments carried out. The value of the control was arbitrarily set to 1, and the other values were derived from it. Three independent experiments were performed, and the values are expressed as the mean ± SD. * denotes *p* < 0.05 vs. the CTRL. ** denotes *p* < 0.01 vs. CTRL. ° denotes *p* < 0.05 vs. H_2_O_2_. °° denotes *p* < 0.01 vs. H_2_O_2_. A Tukey–Kramer comparison test followed the analysis of variance (ANOVA).

Further experiments were performed on cell lines to test the antioxidant potential of PUN. Hydrogen peroxide treatment, in addition to determining the formation and accumulation of free radicals, is also responsible for the induction of lipid peroxidation, a process of degradation of cell membrane lipids caused by free radicals. The oxidative attack of free radicals on lipids causes the breakdown of these molecules in cascade reactions and the formation of malonyldiadehyde (MDA), the presence of which becomes an indicator of oxidative stress and cellular damage [34]. As can be appreciated in Figure 5, in panel a of both boxes, the MDA production is significantly induced by the H_2_O_2_ treatment but reduced significantly by PUN pre-treatment. A similar trend also occurs in astrocytes (panel a, on the right), although again, glial cells appear to be more sensitive than neurons, demonstrating an increase in MDA of 41% compared to that of the neurons. Finally, hydrogen peroxide treatment is also responsible for the accumulation of mitochondrial superoxide, a particularly toxic oxygen reactive species. This result is reported in panel b of both boxes. The accumulation of superoxide anion is slightly greater in astrocytes compared to neurons (not exceeding 20%).

**Figure 6:**
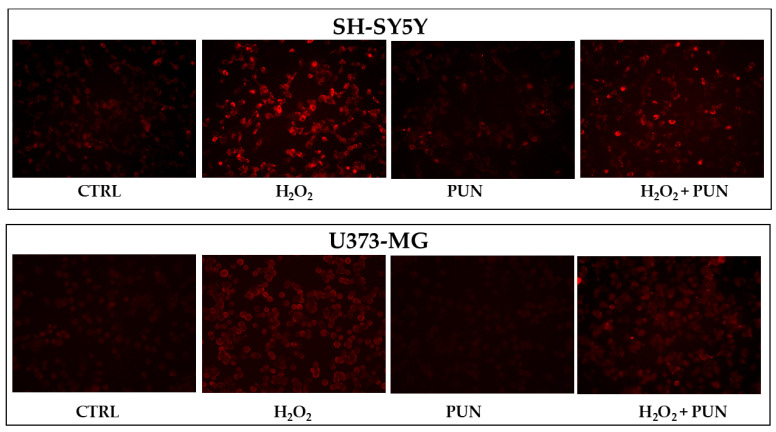
Visualisation of superoxide anion accumulation in neurons and astrocytes. Figure 6 shows the accumulation of superoxide anion, following the specific use of a fluorescent probe. The visualisation was performed using Evos M5000 (Invitrogen by Thermo Fisher Scientific), objective: 20× (0.45 NA/LVD/PHASE), canal: Texas red (TX Red), light: 6.8 as a relative value of the software. At the (**top**) is the panel of neurons, while at the (**bottom**) is that of astrocytes. The treatments carried out on the cells are indicated in the figure. Three independent experiments were performed, but only one representative experiment was shown.

The accumulation of superoxide anion can also be visualised directly on the cells, as represented in Figure 6. Cell visualisation appears to be consistent with numerical data: PUN treatment, followed by hydrogen peroxide exposure, results in greater and equally distributed protection in astrocytes compared to in neurons.

### 3.5. Quantification of Polyphenols and Flavonoids

**Figure 7:**
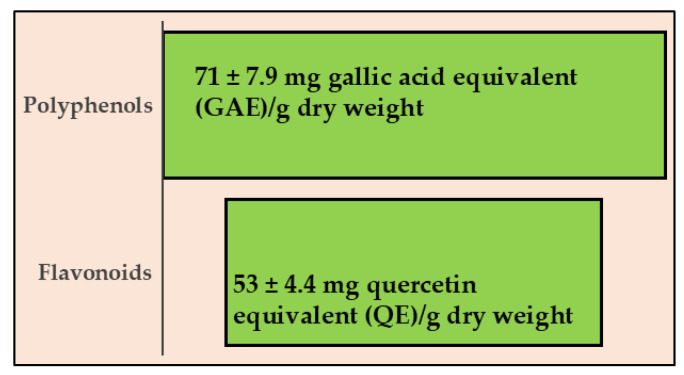
Quantification of polyphenols and flavonoids in PUN.

Since pomegranate is known to contain many polyphenols and flavonoids, we wanted to quantify them in the PUN extract, using the Folin–Ciocalteu colourimetric assay and the aluminium chloride assay, respectively. The results obtained are shown in Figure 7, as can be observed, the amount of polyphenols is greater than that of flavonoids (71 ± 7.9 mg GAE vs. 53 ± 4.4 QE, respectively). These values are comparable to those documented and expressed in the scientific literature [35].

### 3.6. Qualitative and Quantitative Revelation of Tannins

**Figure 8:**
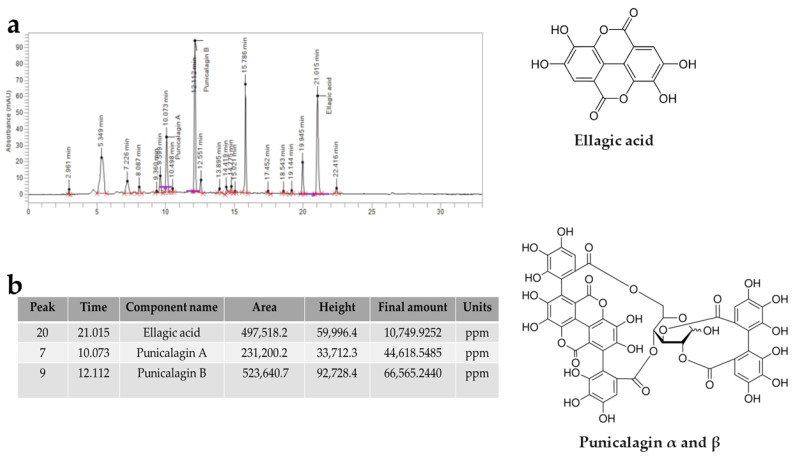
Chromatogram representing the tannins contained in PUN. In panel (**a**), we can observe the tannins present in PUN, detected by HPLC analysis and represented with peaks. Panel (**b**) shows the identification and quantitative data of the tannins involved. Furthermore, the chemical structures of the recognised molecules are also represented in Figure 8. Punicalagin α and β: α-punicalagin, R1 = H and R2 = OH; β-punicalagin, R1 = OH and R2 = H.

Through HPLC analysis, it was possible to qualitatively and quantitatively identify some tannins contained in PUN, as reported in Figure 8. The most represented components were ellagic acid, punicalagin A, and punicalagin B, which derive from the hydrolysis of tannins. The total amount of punicalagin (A + B) is approximately 10 times higher than that of ellagic acid.

#### Identification and Quantification of Anthocyanins

**Figure 9:**
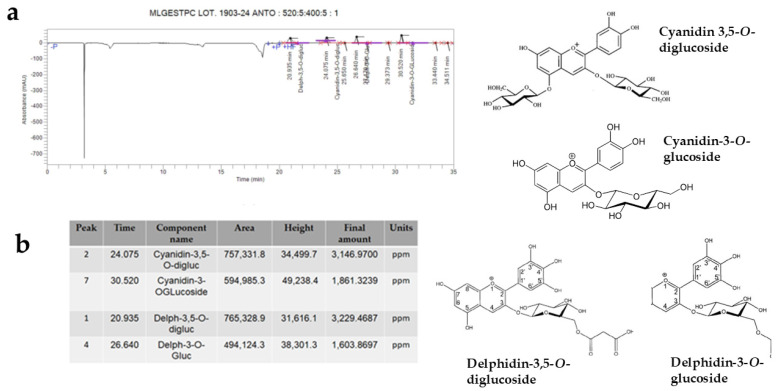
HPLC analysis showed that some anthocyanins (Cyanidin-3,5-*O*-diglucoside, Cyanidin-3-*O*-glucoside, Delphidin-3,5-*O*-diglucoside, Delphidin-3-*O*-glucoside) are present in PUN. Panel (**a**) shows the corresponding peaks, while panel (**b**) highlights the data from the anthocyanins involved. Furthermore, the chemical structures of the recognised molecules are also represented in Figure 9.

Again, through HPLC, it was possible to identify anthocyanins, plant pigments belonging to the large flavonoid family and powerful antioxidants. The anthocyanidins contained in the PUN extract were Cyanidin-3,5-*O*-diglucoside, Cyanidin-3-O-glucoside, Delphinidin-3,5-*O*-diglucoside, and Delphinidin-3-*O*-glucoside. These compounds, in addition to defending cells from the effects of free radicals, can improve cognitive functions, protecting the brain and reducing neurodegeneration [36]. The respective results are shown in Figure 9, while an image of the chemical structure of the main compounds identified in Figure 8 and Figure 9 is shown in Supplementary Figure S1.

### 3.7. Quantification of Non-Extractable Polyphenolic Component (NEPPs)

**Figure 10:**
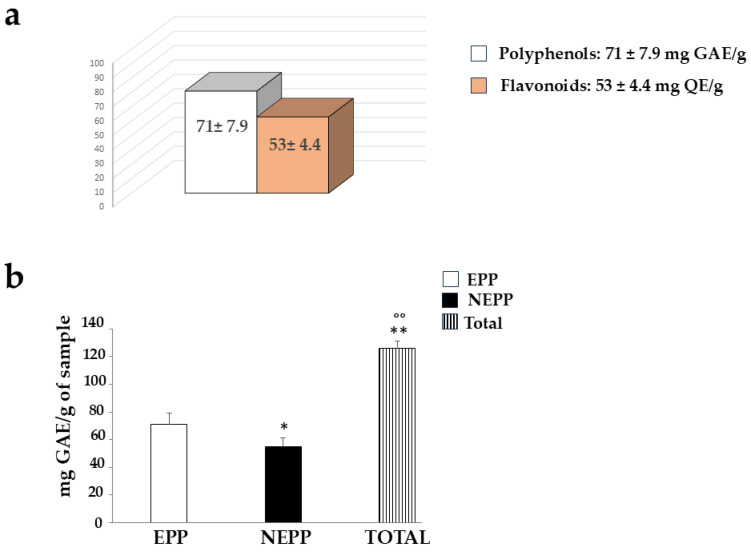
Quantification of non-extractable polyphenols (NEPPs). In panel (**a**), the normally extractable portion of polyphenols (EPPs), quantified via standard methods, and flavonoids is represented. The quantification of NEPPs, released via acid hydrolysis, was obtained through a subsequent Folin–Ciocalteu colourimetric assay, as shown in panel (**b**). Total polyphenols (TOTAL) resulted from the sum of EPPs + NEPPs. Three independent experiments were performed, and the values are expressed as the mean ± SD. * denotes *p* < 0.05 vs. the EPPs. ** denotes *p* < 0.01 vs. EPPs. °° denotes *p* < 0.01 vs. NEPPs. A Tukey–Kramer comparison test followed the analysis of variance (ANOVA).

When the possible presence of NEPPs in PUN was evaluated after employing an extraction method different from that used for normal polyphenolic quantification (both described in the “Section 2”), the results confirmed their presence. This result is described and represented in Figure 10. Panel a shows the same result as Figure 7, while panel b highlights the quantification of NEPPs. An additional fraction of polyphenols (NEPPs, 54.96 ± 6.4 mg GAE) must be added to the freely quantifiable fraction of 71 ± 7.9 mg GAE to yield a significant total amount of polyphenols of 126 ± 5.1 mg GAE)/g dry weight.

## 4. Discussion

The common thread of this scientific work is that PUN exerts a powerful antioxidant effect, and this property was detected not only through direct analysis of the extract but also when PUN was administered to the chosen cell lines. Since it is recognised that pomegranate has a fibrous component and that fibre can exert, among others, beneficial antioxidant effects [15], we decided to analyse the fibre content in pomegranate, as shown in Figure 1. Beta-glucans, which are primarily found in plant cell walls and play both structural and reserve roles, comprise 9.13% of the total fibre in PUN. This fraction shows a predominance of cellulosic material; it represents a modest percentage but is still sufficient to carry out important antioxidant activities, specifically binding polyphenols to their structure [37] and regulating the expression and activity of the main cellular antioxidant enzymes [38,39]. Glucans are markedly outperformed by lignin (insoluble = 22.13%; soluble = 24.95%). Lignin is a complex organic polymer consisting mainly of phenolic compounds and cinnamic acid derivatives. It is present in the cell walls of plants, and its main function is to provide rigidity, hardness, and resistance to the plant, acting as a binder between cellulose fibres [40]. The peel of many fruits is rich in lignin due to the lignification process, which changes the walls of plant cells through lignin deposition. PUN is particularly rich in lignin, as the pomegranate peel is also included in the extract. Lignin also exhibits antioxidant activity due to its polyphenolic structure, which can interact with highly reactive radicals, rendering them inactive or partially reactive [41,42]. Pectin is present in a modest quantity, equal to 0.63%. It is interesting to note that a preponderant percentage (39.95%) is identified as “OTHER”, which can be represented by organic acids, proteins, oligosaccharides of various origins, lipids, waxes, and sterols. This high, variable, and unidentified portion of the fibre could be the key to correctly interpreting the broad antioxidant capacity of PUN: in fact, all tests conducted and shown in Figure 2 (the IC_50_ test, ORAC assay, chelating activity test, and ferric ion reducing antioxidant potential) highlighted this property. In parallel, experiments carried out on cell lines also provided the same results (Figure 4, Figure 5 and Figure 6), highlighting the antioxidant property of PUN. Since we justified this robust antioxidant effect with the presence of polyphenols, we wanted to identify them quantitatively and qualitatively. As can be observed in Figure 7, polyphenols and flavonoids were quantified using the Folin–Ciocalteu colourimetric assay method and the aluminium chloride test, respectively. The results obtained were comparable to those shown in the literature, and the amount of polyphenols was found to be greater than that of flavonoids [71 ± 7.9 mg gallic acid equivalent (GAE)/g dry weight for polyphenols and 53 ± 4.4 mg quercetin equivalent (QE)/g dry weight for flavonoids]. Among the polyphenols, we have identified, through HPLC experiments, some tannins and anthocyanins (Figure 8 and Figure 9, respectively). Anthocyanins belong to the flavonoid class and are the most important pigments of vascular plants [43]. They are responsible for the colours of flowers and fruits and perform numerous functions, including attracting pollinators or promoting seed dispersal [44]. The anthocyanins present in plants are O-glycosides of aglycones (anthocyanidins). Furthermore, anthocyanins and anthocyanidins possess other beneficial functions for human health, including antioxidant activity and anti-inflammatory properties [45]; for example, they are capable of eliminating reactive oxygen and nitrogen species [43]. Tannins are oligomeric polyphenols, and those contained in pomegranate are known as “ellagitannins”, the most significant group of hydrolysable tannins, consisting of over 500 compounds [46]. Ellagitannins release ellagic acid after hydrolysis: the latter carries out various biological activities, including antioxidant, anti-inflammatory, antibacterial, antiviral, anti-tumour, and neuroprotection actions [47]. Among these, the antioxidant potential is based on the reduction of reactive species, the chelation of metal ions, the strengthening of the activity of antioxidant enzymes, and the reduction of lipid peroxidation [48]. When natural products contain many phytochemicals at the same time (flavonoids, anthocyanidins, anthocyanins, phenolic acids, hydrolysable tannins, carotenoids), and this characteristic accentuates some protective effects, such as the antioxidant property, we can identify them as “superfoods” [49]. Superfoods are natural foods exceptionally rich in health-promoting molecules, and pomegranate belongs to this category, containing excellent categories of polyphenols. Globally, polyphenols can interact with each other or with other plant components, resulting in a combined interaction that produces a total synergistic effect greater than the sum of the individual effects [50,51].

**Figure 11:**
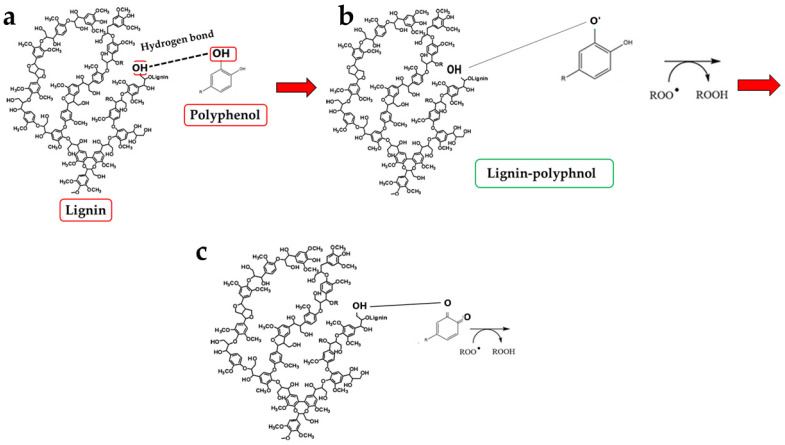
Graphical representation of the polyphenol–carbohydrate bond pattern and its antioxidant properties. Panel (**a**) of the graph shows hydrogen bonding interactions between lignin and polyphenols. Panel (**b**) indicates contact of the lignin–polyphenol complex with a highly reactive species. Finally, panel (**c**) highlights how the reactive species were transformed by polyphenol.

Another key result is the discovery of an additional polyphenolic component, not easily extractable (NEPPs, Figure 10), in pomegranate. When polyphenols spread freely into the extract, they are easily extractable (EPPs) and quantifiable using chemical strategies. The spectrophotometric method used in this study was based on the Folin–Ciocalteu reagent, which oxidises in the presence of polyphenols, forming a blue compound measurable at 750 nm. This method enables the quantification of the total polyphenol content. Chromatographic methods can also be used to ensure a more specific analysis, capable of separating the different polyphenols and identifying the structure through comparison with standards [52]. On the contrary, non-extractable polyphenols are not easily detectable, as they are closely linked to other molecules; are “seized”, not extracted, and accumulate in waste. This occurs due to the chemical nature of dietary polyphenols. Non-extractable polyphenols belong to two categories: (1) They have a high molecular weight, such as some non-extractable proanthocyanidins; (2) They have a low molecular weight and are associated with macromolecules (proteins, dietary fibre), from which they cannot easily separate [53]. This occurs, for example, when polyphenols are linked to polysaccharides or similar molecules by hydrogen bonds. In this case, acid hydrolysis is necessary to release the polyphenols [33]. Optimal extraction conditions have been identified for non-extractable polyphenols and include several variables such as time, temperature, and pH [54]. Therefore, in PUN, the total polyphenol content would be given by the sum of EPPs + NEPPs (71 ± 7.9 + 55 ± 6.4 mg), and it would be equal to 126 ± 14.3 mg gallic acid equivalent (GAE)/g dry weight. These hidden polyphenols could, together with other compounds, not only constitute the percentage of fibre not well identified and generically referred to as “OTHER” (39.95%, Figure 1), but also bind to the countless fibrous carbohydrate monomers via hydrogen bonds [33,55,56]. Bound polyphenols would be constantly susceptible to the performance of antioxidant functions [57]. To date, data on NEPPs content in foods remain limited, but it is important to expand this knowledge, as polyphenols exert several protective effects on human health [58], and limited information may lead to underestimating the effects of some foods. It has been shown that fruit can be a significant source of NEPPs and, even more so, the peel, which is normally considered a waste [53]. The results of this study demonstrate, for the first time, the presence of NEPPs in pomegranate, highlighting that its content could better justify the extraordinary antioxidant capacity recognised in this fruit. If NEPPs were routinely sought and found in plant-based foods, this discovery could favourably and innovatively increase the interest in plant extracts for the management of many diseases. Finally, these results demonstrate that the fibre can exert an antioxidant effect through binding to NEPPs. A model of the antioxidant properties of polyphenols bound to a support molecule, contained within the fibre, is represented in Figure 11. In this graphical model, the support molecule is represented by lignin, which, despite being an organic polymer of phenylpropane units, is present in high amounts in PUN and is rich in -OH groups, which could easily bind polyphenols.

Another innovative result of this study is the different sensitivity of the cell lines to oxidative damage. Indeed, astrocytes suffered the most severe effects compared to those of neurons (Figure 4, Figure 5 and Figure 6) when subjected to oxidative stimulation. In the brain, a neuronal network is closely connected to an astroglial network, and astrocytes support overall brain homeostasis, defending it, collaborating in the construction of the nervous system, and ensuring neurogenesis. Furthermore, astrocytes support the developmental, physiological, and pathological processes of neurons [59]; for example, astrocytes are also known to purify the brain by removing toxins, proteins, and unwanted molecules [60]. If the nervous system were exposed to rapid changes (harmful or beneficial), astrocytes would transform gene expression, morphology, and function, triggering a condition known as “astrocytic reactivity”. Reactive astrocytes can lead to deleterious effects, such as neuroinflammation and synapse impairment, if the stimuli are harmful, or to protective effects, including an anti-inflammatory property, neuroprotective response, and repair of the blood–brain barrier, if the stimuli are beneficial. Today, we know that reactive astrocytes are heterogeneous and not a single uniform entity [61], but we can differentiate two reactive astrocyte phenotypes, known as A1 and A2 [62]. A1 astrocytes lose many normal astrocytic functions, alter synapses, upregulate many genes connected to immune responses, and secrete a soluble neurotoxin that rapidly kills neurons. Therefore, A1 astrocytes could potentially be a harmful phenotype capable of exacerbating the initial stimulus. Conversely, the astrocytic A2 phenotype appears to increase neurotrophic, anti-inflammatory properties; upregulate genes that promote neuronal survival and growth; and support reparative functions, suggesting that they may have a generic protective effect [63]. To date, it is not fully understood how and when the switch from the A1 to the A2 phenotype occurs, nor is the regulation of this phenomenon. This scenario represents the physiological condition in the nervous system. Since the results obtained state unequivocally that the astrocytes appear to suffer oxidative insult more than do the neurons, we can assume that the induced damage causes astrocyte activation and expression of the A1 phenotype.

At the same time, PUN mitigated the effects of oxidative damage, and it could be hypothesised that PUN could be used to extinguish the A1 phenotype.

The continuation of this study should include the use of PUN in vivo. In fact, the study of its chemical characteristics or its administration to cells has not allowed us to evaluate how its bioavailability changes after ingestion and in what amount it reaches the systemic circulation before being eliminated. This information gap represents a major limitation in the knowledge of this extract. In fact, it should not be forgotten that bioavailability depends on several factors, including pH, cooking of the food, health status of the gastrointestinal tract, and the interaction between nutrients [64]. This last factor is important and explains why, in this study, we used the whole extract (PUN) and not its individual components. In fact, as reported in the literature, a plant extract is the set of all active and complementary substances responsible for the induced properties, interacting with each other, and conferring a total synergistic effect greater than the sum of the individual effects [50,51]. Conversely, an in vivo study will consider the bioavailability of the extract after ingestion, and it will be essential to know the effects of each component. The second limitation of this study is related to the greater sensitivity of astrocytes compared to that of neurons to oxidative damage. In fact, the experimental model used was based on the growth of separate cell lines, and astrocytic damage was not directly transmitted to neurons because these two lines were not directly in contact; the use of co-cultures would be interesting in order to evaluate how one cell line behaves in the presence of the other. If the results were similar, it would be interesting to study in detail the A1 phenotype of astrocytes and by what mechanisms PUN would be able to deactivate it; PUN could represent an innovative and successful strategy in the management of neurodegeneration. This hypothesis has not yet been confirmed by further experiments, but the continuation of this scientific study could pursue this direction.

## Data Availability

Data is contained within the article and supplementary materials.

## Supplementary Materials

The following supporting information can be downloaded at: https://www.mdpi.com/article/10.3390/antiox15030276/s1, Supplementary Figure S1: Chemical structures of compounds identified by HPLC. (a) Punicalagin α and β: α-punicalagin, R1 = H and R2 = OH; β-punicalagin, R1 = OH and R2 = H. (b) ellagic acid; (c) Cyanidin-3,5-O-diglucoside; (d) Delphinidin-3-O-glucoside. Several phenolic groups characterise each of these compounds.
