# Supplementary material for: The Significant Antioxidant Effect Exerted by Pomegranate (Punica granatum): The Hidden Polyphenols
Source: Antioxidants (Basel). 2026 Feb 24;15(3):276. doi: 10.3390/antiox15030276 (PMC13024635; doi:10.3390/antiox15030276)
Supplement: Supplementary file 1 [file antioxidants-15-00276-s001.zip › antioxidants-4146976-supplementary.pdf]

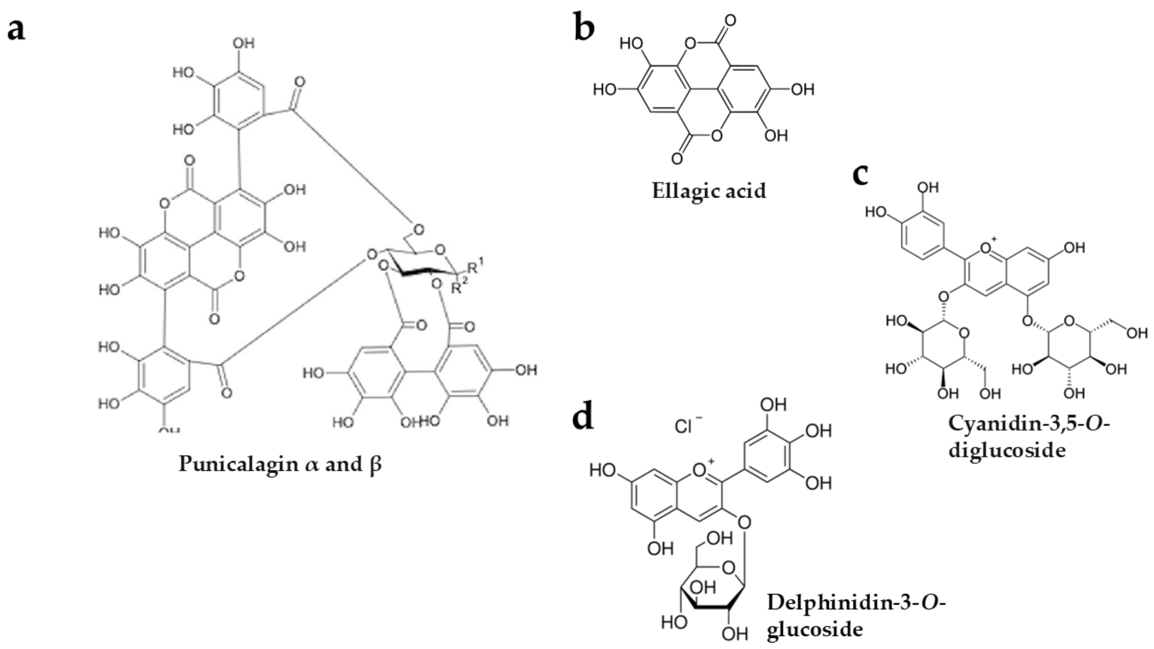

Supplementary Figure S1. Chemical structures of compounds identified by HPLC. a) Punicalagin α and β: α-punicalagin, R1 = H and R2 = OH; β-punicalagin, R1 = OH and R2 = H. b) ellagic acid; c) Cyanidin-3,5-O-diglucoside; d) Delphinidin-3-O-glucoside. Several phenolic groups characterise each of these compounds.
